# A novel splice site variant of the *BBS2* gene in a patient with Bardet-Biedl syndrome

**DOI:** 10.1038/s41439-024-00269-w

**Published:** 2024-03-22

**Authors:** Hasan Azizi, Mortaza Bonyadi, Abbas Rafat

**Affiliations:** 1 Animal Biology Department, Faculty of Natural Sciences, University of Tabriz, Tabriz, Iran; 2 Center of Excellence for Biodiversity, Faculty of Natural Sciences, University of Tabriz, Tabriz, Iran; 3 Department of Animal Science, University of Tabriz, Tabriz, Iran

**Keywords:** Next-generation sequencing, Development

Bardet-Biedl syndrome (BBS) is a genetically heterogeneous ciliopathy with autosomal recessive inheritance and an estimated worldwide prevalence of 1 in 150,000 individuals. Currently, 26 disease-causing genes have been identified for BBS, highlighting its genetic diversity. Here, we report a novel homozygous splice donor variant, NG_009312.2 (NM_031885.5): c.1397+1G>C, in the BBS2 gene. The evidence supports the classification of this variant as “likely pathogenic”.

Bardet-Biedl syndrome (BBS) is a highly pleiotropic ciliopathy characterized by dysfunction of primary motile and immotile cilia. It is primarily inherited in an autosomal recessive manner, although oligogenic (triallelic digenic) inheritance has also been reported^1,2^. BBS is associated with a range of major manifestations, including cystic nephropathy, postaxial polydactyly, developmental delay/cognitive impairment, retinal dystrophy, truncal obesity, and genital anomalies. Minor manifestations of BBS include Hirschsprung’s disease, neurological problems (hypertonia and ataxia), endocrine disorders (such as hypercholesterolemia and type 2 diabetes mellitus), dental disorders, liver involvement, congenital heart disease, and loss or reduction of smell. Clinical diagnosis of BBS requires the presence of four major features or three major features and two minor features^3,4^.

From a genetic perspective, BBS is heterogeneous, and 26 genes associated with BBS have been identified to date. These genes explain the molecular basis of approximately 80% of affected families^4^. Among the genes strongly associated with BBS are MKS1 (4.5%), BBS12 (5%), MKKS (5.8%), BBS9 (6%), BBS2 (8.1%), BBS10 (20%), and BBS1 (23.2%)^5^. In the present report, we describe a BBS patient who was found to have a splice donor variant in the BBS2 gene. To our knowledge, this variant has not been reported previously in patients with BBS Figs. 1 and 2.

**Fig. 1:**
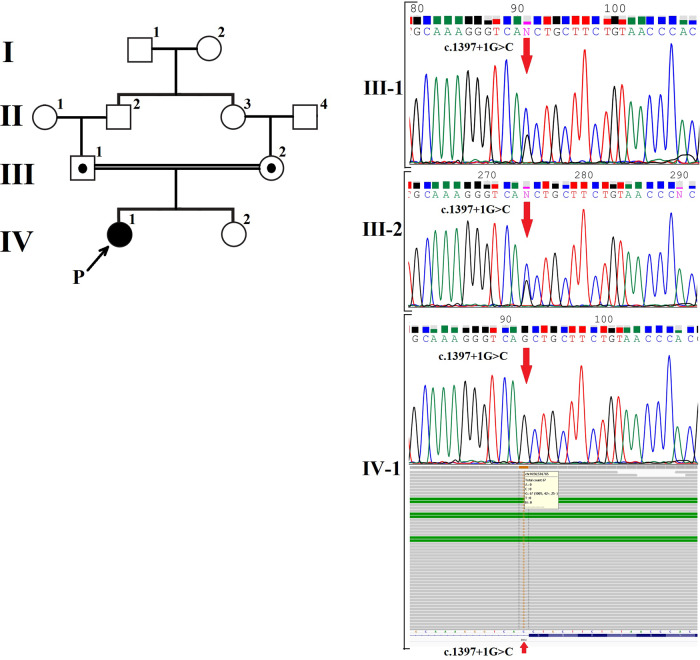
Next-generation sequencing of BBS2 in the patient (proband) revealed a homozygous donor splice site variant at NG_009312.2 (NM_031885.5): c.1397+1G>C (III-1). Sanger sequencing confirmed the presence of a variant in the patient and her parents (the reverse complement strand is shown).

**Fig. 2:**
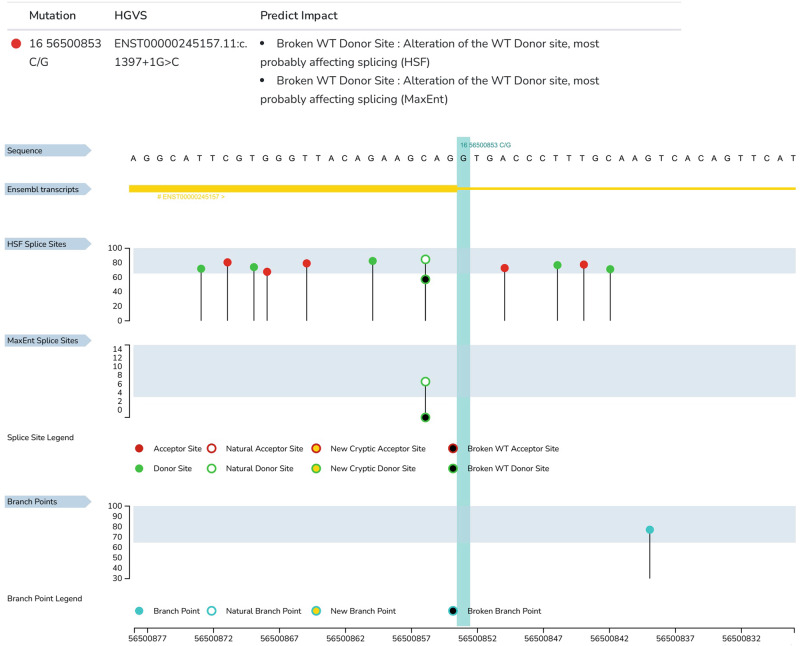
Results of BBS2 c.1397+1G>C variant analysis via the HSF web server. This variant leads to breakdown of the natural splice donor site. As shown by the green circle on the vertical line, there are several splice donor sites around the natural splice donor site. Broken natural splice donor sites can activate these weak splice donor sites. The height of the vertical line represents the strength of the signal.

The patient was a 27-year-old Iranian-Azeri-Turkish woman. She had retinitis pigmentosa, slight intellectual disability, obesity, and ovarian problems (absence of menstrual cycles without medication). The parents of this patient are first cousins. The affected patient was clinically examined by an experienced physician, who confirmed a diagnosis of BBS. Whole-exome sequencing (WES), performed as part of genetic counseling, led to the detection of a homozygous donor splice site variant in the BBS2 gene, NG_009312.2 (NM_031885.5): c.1397+1G>C. We performed Sanger sequencing to confirm the variant in the affected patient and her parents. Sanger sequencing revealed homozygous and heterozygous donor splice site variants of the BBS2 gene, NG_009312.2 (NM_031885.5): c.1397+1G>C, in the patient and her parents, respectively. This novel variant added one nucleotide in exon 11 downstream of the BBS2 gene. This variant was not registered in ClinVar, but substitutions with different bases at the same position have been reported previously^6^. The novel variant was not found in the gnomAD, Turkish Variome, Iranome, ExAC, or 1000G population databases. MutationTaster web server analysis (https://www.mutationtaster.org/index.html) confirmed the deleteriousness of the mutation and the loss of the splice site. The BBS2 gene has 17 exons and encodes a 721-amino acid protein^7^. The c.1397+1G>C mutation in the BBS2 gene is a splice donor site mutation (intron 11). Analysis via the HSF web server (https://hsf.genomnis.com/home) revealed that this variant leads to the breakdown of the wild-type splicing donor site and most likely affects the splicing process. Additionally, the HSF web server revealed that there are several weak splicing donor sites in the vicinity of the altered splice donor site that can be activated. Therefore, the c.1397+1G>C variant can cause intron 11 retention or exon 11 skipping in mature BBS2 mRNA. If splicing leads to the preservation of the full or partial intron 11, the threonine codon at position 467 is replaced by a stop codon, resulting in a truncated protein (p.Thr467Ter). A similar mutation, p.Gln468Ter, has been reported as a likely pathogenic mutation in the ClinVar and dbSNP databases (rs1791390525). Exons 10, 11, and 12 of the BBS2 gene are not frame neutral (with 145, 172, and 130 nucleotides, respectively). Therefore, if splicing causes the deletion of exon 11, after amino acid 408, a sequence of 20 amino acids is altered, and a premature termination codon is created (p.Asp409AlafsX21). Both of these variants most likely lead to mRNA destruction by nonsense-mediated mRNA decay (NMD). Our analysis via the CRYP-SKIP web server (https://cryp-skip.img.cas.cz/) revealed that exon 11 of the BBS2 gene has several cryptic donor and acceptor splice sites, and each of these sites can be activated (P_CR-E_ = 0.40). However, this P_CR-E_ value indicates that exon 11 skipping is more likely to occur. According to the new ClinGen recommendations, this variant is classified as “likely pathogenic”: PVS1_strong and PM2_moderate according to Franklin’s algorithm (https://franklin.genoox.com/; access date: 30 November 2023).

## Data Availability

The relevant data from this Data Report are hosted at the Human Genome Variation Database at 10.6084/m9.figshare.hgv.3373.
